# Utilization of key preventive measures for pregnancy complications and malaria among women in Jimma Zone, Ethiopia

**DOI:** 10.1186/s12889-019-7727-8

**Published:** 2019-11-04

**Authors:** Mariame Ouedraogo, Jaameeta Kurji, Lakew Abebe, Ronald Labonté, Sudhakar Morankar, Kunuz Haji Bedru, Gebeyehu Bulcha, Muluemebet Abera, Beth K. Potter, Marie-Hélène Roy-Gagnon, Manisha A. Kulkarni

**Affiliations:** 1 School of Epidemiology and Public Health, Faculty of Medicine, University of Ottawa, Ottawa, ON Canada; 2 Department of Health, Behavior and Society, Faculty of Public Health, Institute of Health, Jimma University, Jimma, Oromiya Region Ethiopia; 3 Jimma Zonal Health Office, Jimma Zone, Oromiya Region Ethiopia; 4 Department of Population and Family Health, Faculty of Public Health, Institute of Health, Jimma University, Jimma, Oromiya Region Ethiopia

**Keywords:** Pregnant women, Antenatal care, Insecticide-treated nets, Malaria, Ethiopia

## Abstract

**Background:**

In Ethiopia, malaria infections and other complications during pregnancy contribute to the high burden of maternal morbidity and mortality. Preventive measures are available, however little is known about the factors influencing the uptake of maternal health services and interventions by pregnant women in Ethiopia.

**Methods:**

We analyzed data from a community-based cross-sectional survey conducted in 2016 in three rural districts of Jimma Zone, Ethiopia, with 3784 women who had a pregnancy outcome in the year preceding the survey. We used multivariable logistic regression models accounting for clustering to identify the determinants of antenatal care (ANC) attendance and insecticide-treated net (ITN) ownership and use, and the prevalence and predictors of malaria infection among pregnant women.

**Results:**

Eighty-four percent of interviewed women reported receiving at least one ANC visit during their last pregnancy, while 47% reported attending four or more ANC visits. Common reasons for not attending ANC included women’s lack of awareness of its importance (48%), distance to health facility (23%) and unavailability of transportation (14%). Important determinants of ANC attendance included higher education level and wealth status, woman’s ability to make healthcare decisions, and pregnancy intendedness. An estimated 48% of women reported owning an ITN during their last pregnancy. Of these, 55% reported to have always slept under it during their last pregnancy. Analysis revealed that the odds of owning and using ITNs were respectively 2.07 (95% CI: 1.62–2.63) and 1.73 (95% CI: 1.32–2.27) times higher among women who attended at least one ANC visit. The self-reported prevalence of malaria infection during pregnancy was low (1.4%) across the three districts. We found that young, uneducated, and unemployed women presented higher odds of malaria infection during their last pregnancy.

**Conclusion:**

ANC and ITN uptake during pregnancy in Jimma Zone fall below the respective targets of 95 and 90% set in the Ethiopian Health Sector Transformation Plan for 2020, suggesting that more intensive programmatic efforts still need to be directed towards improving access to these health services. Reaching ANC non-users and ITN ownership and use as part of ANC services could be emphasized to address these gaps.

## Background

When well implemented, antenatal care (ANC) can save lives by giving pregnant women the opportunity to receive screening for common pregnancy complications and to be referred for further diagnosis and care [1]. ANC also represents a crucial step for the promotion of healthy habits and behaviours during and after pregnancy [1, 2]. Despite these known benefits, ANC services remain underexploited in most developing countries, including Ethiopia. According to the Ethiopian Demographic Health Survey (EDHS), in 2016, 62% of pregnant women in Ethiopia attended ANC services at least once during their last pregnancy and 32% of women attended four or more ANC visits [3]. Existing evidence also indicates that pregnant women in Ethiopia tend to initiate ANC late in pregnancy, with the majority of women seeking ANC services in the second or third trimester of pregnancy [3]. In addition to its key role in the diagnosis and management of pregnancy complications, ANC services are also essential for the control and prevention of infectious diseases such as malaria, that are known to greatly affect pregnancy outcomes and newborn survival [4].

In Ethiopia, the prevalence of malaria parasitemia in pregnant women is estimated to range between 2.3 and 7.1%, depending on whether areas of unstable and stable endemicity are considered [5]. More recent studies have reported prevalence of malaria in pregnant women ranging between 2.8 and 44.5% [6]. Comparably, the prevalence of placental malaria is estimated at 6.5 and 2.5% in areas of relatively high and low transmission, respectively [5]. Current malaria control and prevention strategies applied by the Ethiopian Federal Ministry of Health include the use of insecticide-treated nets (ITNs) and indoor residual spraying (IRS) in households located in high transmission settings [7]. Given the relatively low prevalence of malaria in Ethiopia, intermittent preventive treatment in pregnancy (IPTp) is not part of the National Malaria Control Strategy (NMCS) [8]. Pregnant women and mothers are targeted during national ITN distribution and IRS campaigns, and health extension workers (HEWs) and other ANC service provision staff are required to distribute an ITN to every pregnant woman attending ANC [7, 9]. ANC service providers and HEWs are also expected to engage in a discussion about ITN possession and use at all visits, and discuss the negative effects that malaria can have on pregnancy [9]. Women who do not attend ANC services may therefore be at a greater risk of malaria and its associated consequences, given their limited access to ITNs and information regarding the relationship between malaria and adverse pregnancy complications.

Few studies have assessed variations in the coverage and usage of ITNs by pregnant women in relation to ANC attendance. A study from Nigeria highlights that attendance to ANC was significantly associated with ownership of bed nets [10]. However, they did not observe any difference in the use of ITNs among women who received ANC and those who did not [10]. Another study in Gabon identified that bed net coverage did not differ according to the number of ANC visits and was not associated with ANC attendance [11]. The lack of relationship was attributed to poor bed net availability within health facilities, and the absence of routine ITN distribution to pregnant women on their first visit [11]. To our knowledge, no published evidence is available on the link between ANC attendance and use of ITNs among pregnant women in Ethiopia.

To attain better maternal health outcomes, it is essential to identify barriers to and facilitators of maternal health services uptake. Herein, we assess the level and determinants of ANC attendance as well as ITN ownership and use within three rural districts of Ethiopia, and examine the relationship between ITN ownership and use, and self-reported malaria infection in pregnant women.

## Methods

### Study design

For the purpose of this study, we used data from a community-based cross-sectional survey that was conducted from October 2016 to January 2017 as part of the baseline evaluation of a larger cluster-randomized controlled trial to address barriers to safe motherhood options in Jimma Zone, Ethiopia (ClinicalTrials.gov identifier: NCT03299491), as described by Ouedraogo et al. (2019) [12]. Only women who had a pregnancy outcome (i.e. live birth, stillbirth, assisted abortion, miscarriage) in the year preceding the survey were eligible to participate.

It was determined that 24 primary health care units (PHCUs) or clusters, and a total of 3840 women would be required for the trial’s baseline evaluation. The sample size calculation for the trial was based on detecting a 17% difference in the primary outcome (skilled birth attendance) between the intervention and control arms. A two-stage sampling strategy was used. We first randomly selected 24 primary health care units (PHCUs) or clusters from the 26 available in the three study districts (Gomma, Kersa, and Seka Chekorsa). From each PHCU catchment area, we then randomly selected 160 eligible women to achieve the target sample size of 3840. Eligible women were randomly selected for face-to-face interviews using a random number generator after conducting a listing exercise with HEWs, village leaders and members of the health development army.

We sought informed consent for each woman after explaining the purpose of the survey and the risks and benefits associated with participation. Of the eligible women, 1.5% women (*N* = 56) refused to participate. No replacement was made for women who refused to participate. Trained interviewers used computer tablets to administer the questionnaire in the local language at the woman’s household. If no woman was available for the interview on the first attempt, the household was revisited on a later date. If no respondent was available after two attempts, the household was replaced by a randomly selected alternate. Survey questions ascertained socio-demographic characteristics of the participants and their maternal health services usage before, during and after pregnancy.

### Study setting

The survey was conducted in three *Woredas* or districts of Jimma Zone: Gomma, Kersa, and Seka Chekorsa. Jimma Zone is situated in Oromiya region, approximately 7 h west by road from Ethiopia’s capital Addis Ababa. Oromiya is one of the regions in Ethiopia where the utilization of maternal and child health services remains suboptimal. The three districts in the study area had a population of approximately 260,000 in 2016. Maternal health services are provided at 26 health centers and 110 rural health posts located in these districts. Nationally, the 2016 EDHS reported that 48% of women who had a live birth in the 5 years before the survey did not attend ANC services [3]. Skilled birth attendance among the 2016 EDHS participants was also low, with only 20% of live births in the 5 years preceding the survey taking place in a health facility [3]. According to the 2015 Ethiopia National Malaria Indicator Survey, 58% of households had at least one ITN and 42% of pregnant women slept under an ITN in Oromiya region [13].

The primary malaria species of epidemiological importance in Ethiopia are *Plasmodium falciparum* and *P. vivax*, accounting for approximately 70 and 30% of the malaria cases, respectively [7]. The risk of malaria infection depends on the altitude, with the greatest risk of infection occurring below 2000 m [7]. In Gomma district, the altitude ranges from 1380 to 1680 m, compared to 1740–2660 m in Kersa district and 1580–2560 m in Seka Chekorsa [14]. The three chosen areas have similar climatic conditions, with a main rainy season occurring from June to August [14]. A short rainy season is also observable in February and March [14]. The incidence of malaria infection is typically greatest during both the rainy seasons while lower infection is observed during the rest of the year [7].

### Study variables

To ascertain ANC attendance in the three districts, women were asked whether they attended ANC during their last pregnancy and how many times they visited a health facility for ANC. Participants who reported not attending ANC were asked why they did not attend. To determine ITN ownership and utilization among the recruited sample, women were asked whether their household owned any ITN during their last pregnancy and how frequently they slept under an ITN during their last pregnancy (never, sometimes, often, always). We re-categorized the utilization of ITN to capture women who always used an ITN during last pregnancy and those who did not always use an ITN (never, sometimes and often). Finally, women were asked to report whether they were diagnosed with malaria during their last pregnancy.

We considered the following socio-demographic variables: age (15–18 years, 19–24 years, 25–34 years, and 35–49 years), marital status (not married, married), ethnic group (Oromo, Amhara, Others), employment status (not employed, self-employed, employed), level of education (no education, primary, secondary, higher), decision-making about health care (husband or family member, self, jointly with husband), exposure to different media sources (not at all, at least once a week, more than once a week), frequency of contact with HEWs (not at all, less than once a month, once or more times a month), household size (≤4, 5–8, ≥9 household members), children in the household (≤3, 4–6, ≥7), reproductive history (i.e. total number of live births, miscarriages, stillbirths, and neonatal death), and last pregnancy intendedness. For employment status, the ‘not employed’ category included individuals who identified as housewives, students and unemployed s, while those identifying as farmers were categorized as self-employed. Following the steps defined by the DHS group, we used a principal component analysis (PCA) to construct a household wealth index combining households’ ownership of various durable assets (e.g. electricity, radio, television, refrigerator, mobile phone)), housing construction (type of materials used for floor, roof and exterior wall), type of toilet facilities, sources of water supply, and type of fuel used for cooking [15]. PCA is a statistical method that reduces a specified number of variables into a smaller number of dimensions or principal components [16]. Briefly, PCA allowed us to extract a principal component from our selected variables as a measure of socio-economic status and derive factor scores for the considered variables. Using the factor scores as weights for each variable, an overall score for each household was subsequently obtained, which can be interpreted as the household-specific wealth score. Based on their wealth score, the households were then divided into five equal quintiles (poorest, poorer, middle, wealthier, wealthiest).

### Statistical analysis

All the data were synchronized to a cloud server using Open Data Kit and we exported CSV files for data management and analysis. We performed all analyses using SAS statistical software version 9.4. We assessed the distribution of each variable through frequency tables. We used chi-square and Fisher’s exact tests, as appropriate, to explore associations between the variables and determine differences in proportions. Differences in means were assessed using t-tests. We considered a two-sided *p*-value of 0.05 as the level of statistical significance.

Using unadjusted and adjusted logistic regression models, we generated odds ratio (ORs) and corresponding 95% confidence intervals to identify significant predictors of first ANC visit attendance. We considered all the aforementioned socio-demographic characteristics (i.e. age, marital status, employment status, level of education, decision-making about health care, exposure to television and radio, frequency of contact with HEWs, reproductive history, and last pregnancy intendedness) as potential predictors of ANC attendance in univariable analyses, and retained all significant variables (*p* > 0.05) in the multivariable models. We conducted a sub-group analysis among women who attended any ANC visit, to identify whether the participants who attended at least four ANC visits differed from women who attended three or fewer ANC visits with regards to their socio-demographic characteristics.

We used multivariable logistic regression analyses to assess the relationship between ANC attendance (no attendance, at least one visit) and ITN ownership and utilization. We considered age, marital and occupation status, education status, household wealth, and whether the dwelling was sprayed with insecticide in the last year as potential confounders and effect modifiers. Confounding was assessed by looking at the strength of the association between each variable, and ANC attendance and ITN ownership and utilization. Effect modification was investigated by introducing interaction terms between main predictors and potential effect modifiers into the regression models. We similarly assessed the main predictors of malaria infection during pregnancy using logistic regression models, considering socio-demographic factors, ITN utilization and IRS as potential predictors. For the analyses of ITN ownership and use, and malaria in pregnancy, we performed subgroup analyses considering only women living within the catchment area of PHCUs at an altitude below 2000 m, where the risk of malaria is known to be higher.

Our analysis considered the complex cross-sectional survey sampling design, wherein women were clustered within PHCUs, as failure to do so can lead to incorrect inferences [17]. We therefore incorporated clustering of the data in all the analyses through logistic regressions with random intercept models, using the PROC GLIMMIX function in SAS 9.4. We also ran model diagnostics, including assessment of the distribution of the residuals and random effects.

## Results

### Characteristics of the study participants

A total of 3784 women agreed to take part in the cross-sectional survey (Table 1). The response rate was of 98.5%. Half of the participating women across the three districts were aged between 25 and 34 years (50.5%). Oromo was the most dominant ethnic group, representing 94% of participants across all districts. Nearly all of the participating women were married (97.7%) and unemployed (78.2%). Kersa district had the highest proportion of women who reported not having a formal education (67.9%) followed by Seka Chekorsa (53.9%) and Gomma (47.2%). The majority of women reported making decisions regarding health care jointly with their husband (59.2%). Kersa had the largest proportion of participants in the poorest wealth quintile (35.9%). An important proportion of the participants were not exposed to any media sources (i.e. newspapers/magazines (93%), radio (46%), and/or television (88%)). Close to 11% of the women reported being visited frequently by an HEW during their last pregnancy (defined as one or more visits per month).

**Table 1:** Characteristics of the women who participated in the cross-sectional survey conducted in three districts of Jimma Zone, Ethiopia, from October 2016 to January 2017 ^a^

	Total (*N* = 3784)	Gomma (*N* = 1402)		Kersa (*N* = 1110)		Seka Chekorsa (*N* = 1272)	
	%	N	%	N	%	N	%
Maternal age (years)							
15–18	7.5	95	6.8	80	7.2	110	8.6
19–24	25.5	345	24.6	264	23.8	356	27.9
25–34	50.5	736	52.5	559	50.4	615	48.3
35–49	16.5	226	16.1	207	18.6	191	15.0
Ethnic group							
Oromo	93.7	1262	90.0	1065	95.9	1220	95.9
Amhara	1.9	58	4.1	4	0.4	11	0.9
Other	4.3	82	5.8	41	3.7	41	3.2
Education level							
No education	55.5	661	47.2	754	67.9	686	53.9
Primary^b^	31.0	470	33.5	273	24.6	430	33.8
Secondary^c^	12.7	259	18.5	77	6.9	146	11.5
Higher	0.7	12	0.9	6	0.5	10	0.8
Marital Status							
Married	97.7	1348	96.2	1098	98.9	1252	98.5
Not married	2.3	54	3.8	12	1.1	19	1.5
Employment status							
Not Employed^d^	78.2	1132	80.7	845	76.1	981	77.1
Self-employed^e^	19.2	211	15.1	255	22.9	260	20.4
Employed	2.6	59	4.2	10	0.9	31	2.4
Wealth							
Poorest	20.0	89	6.3	399	35.9	270	21.2
Poorer	21.3	252	17.9	227	20.4	326	25.6
Middle	18.6	222	15.8	213	19.2	269	21.1
Wealthier	20.1	309	22.0	186	16.7	265	20.8
Wealthiest	20.0	530	37.8	85	7.66	142	11.2
Frequency of reading newspaper/ magazine							
Not at all	92.8	1268	90.4	1065	95.9	1180	92.7
At least once a week	5.8	114	8.1	35	3.1	73	5.7
More than once a week	1.3	20	1.4	10	0.9	19	1.5
Frequency of listening to the radio							
Not at all	45.6	622	44.4	534	48.1	569	44.7
At least once a week	26.5	422	30.1	272	24.5	309	24.3
More than once a week	27.9	358	25.5	304	27.4	394	30.9
Frequency of watching the TV							
Not at all	88.8	1070	76.3	1066	96.0	1226	96.4
At least once a week	5.2	150	10.7	26	2.3	22	1.7
More than once a week	5.9	182	12.9	18	1.6	24	1.9
Frequency of contact with HEW							
Not at all	66.4	856	61.0	856	77.1	802	63.0
Less than once a month	23.0	348	24.8	200	18.0	323	25.4
One or more times a month	10.5	198	14.1	54	4.8	147	11.5
Decision Making about healthcare							
Self	12.2	170	12.1	120	10.8	171	13.4
Jointly	59.2	883	62.9	628	56.6	728	57.2
Husband	27.9	336	23.9	355	31.9	366	28.7
Family member	0.7	13	0.9	7	0.6	7	0.2
Household size							
≤ 4	34.0	504	35.9	329	29.6	454	36.7
5–8	56.2	794	56.6	629	56.7	702	55.2
≥ 9	9.8	104	7.4	152	13.7	116	9.1
Number of children per household							
≤ 3	56.2	848	60.5	553	49.8	725	57.0
4–6	38.7	506	36.1	465	41.9	493	38.7
≥ 7	5.1	48	3.4	92	8.3	54	4.25
Reproductive history							
Gravidity (mean ± SD)^f^	3.70 (±2.34)	3.51 (±2.25)		4.15 (±2.47)		3.52 (±2.27)	
Live birth							
1	1.6	32	2.3	7	0.6	21	1.6
> 1	98.4	1370	97.7	1103	99.4	1251	98.3
Induced abortion							
0	94.1	1295	92.4	1058	95.3	1210	95.1
≥ 1	5.9	107	7.6	52	4.7	62	4.9
Miscarriage							
0	97.4	1347	96.1	1088	98.0	1250	98.3
≥ 1	2.6	55	3.9	22	2.0	22	1.7
Stillbirth							
0	95.5	1314	93.7	1066	96.0	1234	97.0
≥ 1	4.5	88	6.3	44	3.9	38	2.9
Malaria							
Malaria infection during pregnancy							
Yes	1.4	28	2.0	9	0.8	18	1.4
No	98.5	1374	98.0	1101	99.2	1254	98.6
Bed net ownership							
Yes	47.6	874	62.3	484	43.6	444	34.9
No	52.4	528	37.6	626	56.4	828	65.1
Bed net use							
Never	53.3	547	39.0	635	57.2	833	65.6
Sometimes	8.5	153	10.9	83	7.5	85	6.7
Often	11.8	182	13.0	147	13.2	119	9.4
Always	26.4	520	37.1	245	22.1	232	18.3

Notes: ^a^ Numbers are rounded and may not add up to exactly 100%; ^b^considers women who completed all or some primary school years; ^c^ considers women who completed all or some secondary school years; ^d^ the unemployed category contains women who were unemployed, housewives or students; ^e c^ the self-employed category contains women who are farmers and/or traders; ^f^ Defined as the number of times a woman has been pregnant

Analysis of the reproductive history of the participants revealed that, on average, the number of pregnancies per woman ranged between three and four. In Gomma, 7.6% of the women had at least one induced abortion, compared to 4% in Kersa and 5% in Seka Chekorsa. The proportion of women with a history of prior miscarriage in each district was low. Across the three districts, 4.5% of the women had history of at least one stillbirth.

### Antenatal care attendance determinants and barriers

Of the 3784 women who participated in the survey, 84.2% reported attending at least one ANC visit during their last pregnancy (90.3, 75.5 and 85.2% in Gomma, Kersa and Seka Chekorsa, respectively). Overall, 47.0% of the participants reported seeking four or more ANC visits during their pregnancy (54.2, 38.1 and 46.4% in Gomma, Kersa and Seka Chekorsa, respectively).

We observed that 16.0% of women (*N* = 597) did not attend ANC services. The main reasons for not seeking ANC were a perceived lack of necessity of ANC services (45%) or time (14%), far distance to the closest health center (22%), and the absence of transportation (13%). Other non-negligible barriers included the absence of husband’s support (8%), sickness or complications related to pregnancy (7%), poor service within the health facility (5%), and the unavailability of childcare (5%). Thirty-two participants did not provide reasons for not attending ANC.

In the multivariable analysis, education (primary education AOR: 1.47, 95% CI: 1.16–1.87, secondary or higher education AOR: 2.38, 95% CI:1.49–3.82, compared to no formal education) and wealth (wealthiest quintile AOR: 4.29, 95% CI: 2.89–6.35, compared to poorest quintile) were identified as the most important predictors of attending at least one ANC visit (Table 2). Frequent contacts with an HEW resulted in higher odds of ANC attendance. Similarly, women who were able to make decisions regarding their healthcare by themselves or jointly with their partners, women who experienced health problems during their last pregnancy, and women who had wanted their last pregnancy were 26, 58 and 68% more likely to attend at least one ANC visit, respectively. However, women who were of an ethnic group other than Oromo or Ahmara were 42 less likely to attend at least one ANC visit compared to women who were part of the largest ethnic group (i.e. Oromo). A significant positive association was found between frequent television use and ANC attendance; however, this was subject to small sample sizes.

**Table 2:** Association between maternal characteristics and attending at least one antenatal care visit among women who experienced a pregnancy outcome in the past year in three districts of Jimma Zone, Ethiopia, 2016^a^

	Attended at least one ANC visit N (%)	Did not attend ANC N (%)	*P* value for the overall difference among the categories	Factors associated with attending at least one antenatal care visit, univariate analysis OR (95% CI)	Factors associated with attending at least one antenatal care visit, multivariable analysis AOR (95% CI)^g^
Maternal age (years)					
15–18	231 (7.2)	54 (9.0)	**< 0.0001**	Reference	Reference
19–24	864 (27.1)	101 (16.9)		**1.83 (1.25–2.67)**	1.27 (0.84–1.91)
25–34	1594 (50.0)	316 (52.9)		1.01 (0.72–1.42)	1.26 (0.85–1.88)
35–49	498 (15.6)	126 (21.1)		0.884 (0.58–1.23)	1.33 (0.84–2.10)
Ethnic group					
Oromo	2995 (93.9)	552 (92.5)	**0.0113**	Reference	Reference
Amhara	66 (2.1)	7 (1.2)		1.11 (0.49–2.50)	0.85 (0.35–1.95)
Other	126 (3.9)	38 (6.4)		**0.56 (0.37–0.84)**	**0.58 (0.38–0.89)**
Education level					
No education	1660 (52.1)	441 (73.8)	**< 0.0001**	Reference	Reference
Primary^b^	1041 (32.6)	132 (22.1)		**1.92 (1.55–2.39)**	**1.47 (1.16–1.87)**
Secondary^c^ or Higher^d^	486 (15.2)	24 (4.0)		**4.57 (2.96–7.05)**	**2.38 (1.49–3.82)**
Employment Status					
Not Employed^e^	2510 (78.7)	448 (75.0)	**0.0004**	Reference	Reference
Self-employed^f^	583 (18.3)	143 (23.9)		**0.78 (0.63–0.97)**	0.79 (0.63–1.01)
Employed	94 (2.9)	6 (1.0)		**2.74 (1.16–6.45)**	1.68 (0.69–4.09)
Wealth					
Poorest	544 (17.1)	214 (35.8)	**0.0001**	Reference	Reference
Poorer	649 (20.4)	156 (26.1)		**1.45 (1.13–1.87)**	**1.53 (1.17–1.99)**
Middle	614 (19.3)	90 (15.1)		**2.319 (1.74–3.08)**	**2.52 (1.87–3.39)**
Wealthier	665 (20.8)	95 (15.9)		**2.27 (1.71–3.03)**	**2.37 (1.76–3.19)**
Wealthiest	715 (22.4)	42 (7.0)		**4.96 (3.39–7.23)**	**4.29 (2.89–6.35)**
Frequency of reading newspaper/ magazine					
Not at all	2936 (92.1)	577 (96.6)	**0.0006**	Reference	Reference
At least once a week	203 (6.4)	19 (3.2)		**1.84 (1.12–3.00)**	0.67 (0.38–1.18)
More than once a week	48 (1.51)	1 (0.2)		**11.64 (1.58–85.81)**	2.48 (0.31–19.56)
Frequency of listening to the radio					
Not at all	1373 (43.1)	352 (58.9)	**< 0.0001**	Reference	Reference
At least once a week	879 (27.6)	124 (20.8)		**1.76 (1.39–2.22)**	1.27 (0.99–1.64)
More than once a week	935 (29.3)	121 (20.3)		**1.97 (1.56–2.49)**	**1.28 (1.00–1.6**6)
Frequency of watching the TV					
Not at all	2783 (87.3)	579 (96.9)	**< 0.0001**	Reference	Reference
At least once a week	185 (5.8)	13 (2.2)		**2.30 (1.29–4.21)**	1.39 (0.74–2.62)
More than once a week	219 (6.9)	5 (0.8)		**6.18 (2.50–15.28)**	**2.98 (1.15–7.74)**
Frequency of contact with HEW					
Not at all	2053 (64.4)	461 (77.2)	**< 0.0001**	Reference	Reference
Less than once a month	773 (24.2)	98 (16.4)		**1.61 (1.26–2.05)**	**1.69 (1.15–2.48)**
One or more times a month	361 (11.3)	38 (6.4)		**1.86 (1.29–2.67)**	**1.66 (1.27–2.13)**
Decision-making about healthcare					
Husband or family member	874 (23.1)	210 (5.5)	**0.0086**	Reference	Reference
Self	385 (12.1)	76 (12.7)		1.16 (0.86–1.57)	1.10 (0.81–1.51)
Jointly with husband	1928 (60.5)	311 (52.1)		**1.45 (1.21–1.81)**	**1.26 (1.02–1.56)**
Household size					
≤ 4	1155 (36.2)	132 (22.1)	**< 0.0001**	Reference	Reference
5–8	1751 (54.9)	374 (62.6)		**0.54 (0.44–0.67)**	0.87 (0.63–1.19)
≥ 9	281 (8.8)	91 (15.2)		**0.43 (0.31–0.58)**	0.75 (0.45–1.26)
Number of children per household					
≤ 3	1882 (59.0)	244 (40.9)	**< 0.0001**	Reference	Reference
4–6	1159 (36.4)	305 (51.1)		**0.51 (0.42–0.62)**	0.74 (0.55–1.00)
≥ 7	146 (4.6)	48 (8.0)		**0.47 (0.33–0.68)**	0.76 (0.41–1.39)
Health problems during last pregnancy					
No	2491 (78.2)	509 (85.2)	**0.0050**	Reference	Reference
Yes	694 (21.8)	88 (14.7)		**1.57 (1.22–2.02)**	**1.58 (1.22–2.05)**
Number of live birth					
0–1	742 (23.7)	79 (13.4)	**< 0.0001**	Reference	Reference
2–4	1424 (45.4)	229 (38.8)		**0.67 (0.52–0.88)**	0.97 (0.70–1.36)
≥ 5	968 (30.9)	282 (47.8)		**0.40 (0.31–0.53)**	0.84 (0.55–1.29)
Number of child-deaths					
None	2734 (85.8)	485 (82.1)	**0.0059**	Reference	Reference
≥ 1	453 (14.2)	112 (18.8)		**0.78 (0.61–0.98**)	1.04 (0.79–1.35)
Intended pregnancy					
No	967 (30.3)	309 (51.7)	**< 0.0001**	Reference	Reference
Yes	2220 (69.6)	288 (48.2)		**2.18 (1.81–2.63)**	**1.68 (1.38–2.06)**

*Abbreviations*: *CI* Confidence interval, *OR* Odds ratio, *AOR* Adjusted odds ratio

Notes: ^a^ Numbers are rounded and may not add up to exactly 100%; ^b^considers women who completed all or some primary school years; ^c^ considers women who completed all or some secondary school years; ^d^ secondary and higher education were combined given model conversion issues; ^e^ the unemployed category contains women who were unemployed, housewives or students; ^f^ the self-employed category contains women who are farmers and/or traders; ^g^ AOR and corresponding 95% CI were obtained from a multivariate model, including variables with a significant univariate test at *p*-value cut-off point of 0.05

Bold text indicates significant results at the level of *p*-value <0.05

Respondents had higher odds of attending four or more ANC visits if they had completed some secondary (AOR: 1.90, 95% CI: 1.46–2.47) or higher (AOR: 5.24, 95% CI: 1.53–17.95) education, relative to those who reported no education completion (Table 3). Wealth was also identified as an important determinant of attending four or more ANC visits, although only the odds ratios for respondents that were from the wealthiest quintile were significantly different from the poorest group (AOR:1.64, 95% CI:1.27–2.12). Women who made decisions about their health by themselves were 33% more likely to attend four or more ANC visits, while women who decided jointly with their partner were 32% more likely to seek four or more ANC visits. Women who were part of a household with more than seven children were 46% less likely to seek ANC four or more times than women who had three or fewer children part of their household. Desiring the last pregnancy was also a significant predictor, with an AOR of 1.37 (95% CI: 1.17–1.62). However, we found that women who had a prior stillbirth were less likely to attend four or more ANC visits.

**Table 3:** Association between maternal characteristics and attending four antenatal care visits among women who experienced a pregnancy outcome in the past year in three districts of Jimma Zone, Ethiopia, 2016^a^

	Attended four antenatal care visits N (%)	Did not attend four antenatal care visits N (%)	*P* value for the overall difference among the categories	Factors associated with attending four antenatal care visits, univariate analysis OR (95% CI)	Factors associated with attending four antenatal care visits, multivariable analysis AOR (95% CI)^d^
Maternal age (years)					
15–18	123 (6.9)	108 (7.6)	**0.0037**	Reference	Reference
19–24	530 (29.8)	334 (23.6)		**1.41 (1.05–1.89)**	1.24 (0.91–1.68)
25–34	863 (48.6)	731 (51.7)		1.01 (0.78–1.36)	1.04 (0.76–1.42)
35–49	259 (14.6)	239 (16.9)		0.96 (0.69–1.31)	1.01 (0.69–1.46)
Education level					
No education	854 (48.1)	806 (57.1)	**< 0.0001**	Reference	Reference
Primary^b^	564 (31.8)	477 (33.8)		1.11 (0.95–1.30)	0.99 (0.83–1.18)
Secondary^e^	332 (18.7)	126 (8.9)		**2.47 (1.96–3.11)**	**1.90 (1.46–2.47)**
Higher	25 (1.4)	3 (0.2)		**8.38 (2.50–28.07)**	**5.24 (1.53–17.95)**
Wealth					
Poorest	268 (15.1)	276 (19.5)	**< 0.0001**	Reference	Reference
Poorer	312 (17.6)	337 (23.8)		0.92 (0.73–1.16)	0.92 (0.73–1.17)
Middle	344 (19.4)	270 (19.1)		1.27 (1.00–1.61)	1.27 (0.99–1.61)
Wealthier	371 (20.9)	294 (20.8)		1.26 (1.00–1.59)	1.19 (0.94–1.51)
Wealthiest	480 (27.0)	235 (16.6)		**2.01 (1.58–2.56)**	**1.64 (1.27–2.12)**
Frequency of reading newspaper/ magazine					
Not at all	1618 (91.1)	1318 (93.3)	**0.0278**	Reference	Reference
At least once a week	128 (7.2)	75 (5.3)		**1.37 (1.01–1.84)**	0.76 (0.54–1.07)
More than once a week	29 (1.6)	19 (1.3)		1.28 (0.71–2.32)	**0.38 (0.18–0.75)**
Frequency of watching the TV					
Not at all	1490 (83.9)	1293 (91.6)	**< 0.0001**	Reference	Reference
At least once a week	133 (7.5)	52 (3.7)		**2.15 (1.53–3.00)**	**1.60 (1.12–2.30)**
More than once a week	152 (8.5)	67 (4.7)		**1.91 (1.41–2.59)**	1.25 (0.88–1.79)
Frequency of contact with HEW					
Not at all	1098 (61.8)	955 (67.6)	**0.0027**	Reference	Reference
Less than once a month	483 (27.2)	290 (20.5)		**1.42 (1.19–1.68)**	**1.37 (1.15–1.64)**
One or more times a month	194 (10.9)	167 (11.8)		0.95 (0.76–1.20)	0.88 (0.69–1.11)
Decision-making about healthcare					
Husband or family member	426 (24.0)	448 (31.7)	**0.0002**	Reference	Reference
Self	221 (12.4)	164 (11.6)		**1.39 (1.09–1.77)**	**1.33 (1.03–1.70)**
Jointly	1128 (63.5)	800 (56.6)		**1.46 (1.24–1.72)**	**1.32 (1.11–1.55)**
Household size					
≤ 4	692 (38.9)	463 (32.8)	**0.0021**	Reference	Reference
5–8	936 (52.7)	815 (57.7)		**0.77 (0.66–0.89)**	0.94 (0.76–1.16)
≥ 9	147 (8.3)	134 (9.5)		**0.74 (0.57–0.97)**	1.24 (0.81–1.92)
Number of children per household					
≤ 3	1097 (61.8)	785 (55.6)	**0.0009**	Reference	Reference
4–6	612 (34.5)	547 (38.7)		**0.80 (0.69–0.93)**	0.91 (0.72–1.14)
≥ 7	66 (3.7)	80 (5.6)		**0.61 (0.43–0.85)**	**0.54 (0.31–0.92)**
Health problems during last pregnancy					
No	1368 (77.1)	1125 (79.7)	**0.0385**	Reference	Reference
Yes	407 (22.9)	287 (20.3)		1.18 (1.00–1.41)	1.17 (0.98–1.40)
Number of live birth					
0–1	479 (26.9)	316 (22.4)	**0.0147**	Reference	Reference
2–4	789 (44.4)	635 (44.9)		**0.73 (0.59–0.88)**	1.08 (0.87–1.36)
≥ 5	507 (28.5)	461 (32.6)		**0.81 (0.68–0.97)**	1.29 (0.87–1.36)
Prior stillbirth					
No	1708 (96.2)	1330 (94.2)	**0.0217**	Reference	Reference
Yes	67 (3.8)	82 (5.8)		**0.63 (0.45–0.88)**	**0.65 (0.46–0.92)**
Intended pregnancy					
No	467 (26.3)	500 (35.4)	**< 0.0001**	Reference	Reference
Yes	1308 (73.7)	912 (64.6)		**1.54 (1.32–1.79)**	**1.37 (1.17–1.62)**

*Abbreviations*: *CI* Confidence interval, *OR* Odds ratio, *AOR* Adjusted odds ratio

Notes: ^a^ Numbers are rounded and may not add up to exactly 100%; ^b^considers women who completed all or some primary school years; ^c^ considers women who completed all or some secondary school year; ^d^ AOR and corresponding 95% CI were obtained from a multivariate model, including variables with a significant univariate test at *p*-value cut-off point of 0.05

Bold text indicates significant results at the level of *p*-value <0.05

### Malaria infection and insecticide-treated net ownership and utilization during pregnancy

We observed low rates of malaria infection during pregnancy in our sampled group with 2.0% (*N* = 28), 0.8% (*N* = 9), and 1.4% (*N* = 18) of women reporting they were diagnosed with malaria during their last pregnancy in Gomma, Kersa, and Seka Chekorsa, respectively. Fifty-one percent of the malaria cases were reported by women in Gomma. The difference in malaria prevalence across the three districts was not statistically significant. Out of the total sample, 58% (*N* = 2195) of survey respondents were from areas below 2000 m. Seventy-one percent of reported malaria cases were detected in women from areas at an altitude below 2000 m. The prevalence of self-reported malaria in women living in areas below 2000 m was 1.9% (*N* = 18) in Gomma, 1.0% (*N* = 8) in Kersa and 2.5% (*N* = 12) in Seka Chekorsa (See Additional file 1: Table S1). The difference in malaria infection rates between the districts was not statistically significant (*p* = 0.096).

ITN ownership varied across the three districts with 62.3% of surveyed women in Gomma, 43.6% in Kersa, and 34.9% in Seka Chekorsa reporting that they possessed an ITN during their last pregnancy. ITN use was below the level of ITN ownership with 37.1% of women in Gomma, 22.1% in Kersa and 18.3% reportingto have always used their ITN. Considering women from areas where malaria is potentially endemic, we found that 75.5% in Gomma, 56.9% in Kersa and 60.2%in Seka Chekorsa owned a ITN during their last pregnancy. The utilization of ITNs was lower with 45.4, 29.1 and 31.1% of women who owned at least one ITNreported to have always used it during their last pregnancy in Gomma, Kersa and Seka Chekorsa, respectively (See Additional file 1: Table S1).

### Antenatal care attendance and insecticide-treated net ownership and use

After adjusting for the main potential confounders and considering effect modifiers such as maternal age, ethnicity, education level, occupation status, wealth, household size, and IRS, we found that participants who attended at least one ANC had 2.07 (95% CI: 1.62–2.63) times the odds of having owned an ITN during their last pregnancy (Table 4). Similarly, we found that women who attended at least one ANC visit were 73% (AOR: 1.73 (95% CI: 1.32–2.27) more likely to have always used an ITN during their last pregnancy (Table 5). Our subgroup analyses considering only the women from areas where malaria is potentially endemic (altitude below 2000 m) suggested that women who attended at least one ANC visit were 2.41 (95% CI: 1.82–3.19) times more likely to own a net and 1.75 (1.29–2.38) times more likely to report always having slept under a net during their last pregnancy (See Additional file 1: Tables S2 & S3).

**Table 4:** Association between antenatal care attendance and the ownership of insecticide-treated nets among women who experienced a pregnancy outcome in the past year in three districts of Jimma Zone, Ethiopia, 2016

	Owned a net N (%)	Did not own a net N (%)	*P* value for the overall difference among the categories	Association with owning an ITN univariate analysis OR (95% CI)	Association with owning an ITN, multivariable analysis AOR (95% CI) ^a^
ANC attendance					
No	177 (9.8)	420 (21.2)	**< 0.0001**	Reference	Reference
At least once	1625 (90.2)	1562 (78.8)		**2.13 (1.68–2.68)**	**2.07 (1.62–2.63)**

*Abbreviations*: *ANC* Antenatal care, *CI* Confidence interval, *ITN* Insecticide-treated net, *OR* Odds ratio, *AOR* Adjusted odds ratio

^a^ Adjusting for main confounders: maternal age, ethnicity, education level, occupation status, wealth, household size and indoor residual spraying

Bold text indicates significant results at the level of *p*-value <0.05

**Table 5:** Association between antenatal care attendance and the utilization of insecticide-treated nets among women who experienced a pregnancy outcome in the past year in three districts of Jimma Zone, Ethiopia, 2016

	Always used a net N (%)	Did not always use a net N (%)	*P* value for the overall difference among the categories	Association with always using an ITN, univariate analysis OR (95% CI)	Association with always using an ITN, multivariable analysis AOR (95% CI)^a^
ANC attendance					
None	85 (8.5)	512 (18.4)	**< 0.0001**	Reference	Reference
At least once	912 (91.5)	2275 (81.6)		**1.86 (1.43–2.43)**	**1.73 (1.32–2.27)**

*Abbreviations*: *ANC* Antenatal care, *CI* Confidence interval, *ITN* Insecticide-treated net, *OR* Odds ratio, *AOR* Adjusted odds ratio

^a^ Adjusting for main confounders: maternal age, ethnicity, education level, occupation status, wealth, household size, indoor residual spraying

Bold text indicates significant results at the level of *p*-value <0.05

### Risk factors of malaria infection in pregnancy

Women who reported that they always used an ITN during their last pregnancy were less likely to report experiencing a malaria infection, although the association was not statistically significant (AOR: 0.87 (95% CI: 0.44–1.72)) (Table 6). High maternal age (25–49 years compared to younger age groups) and primary education level (compared to no education) were significantly associated with lower odds of malaria infection during pregnancy. Women who were self-employed were 2.8 times more likely to have a malaria infection during their past pregnancy than women who were unemployed. In the analyses with women living in areas where malaria is potentially endemic, we found that employment was significantly associated with self-reported malaria infection. Compared to unemployed women, self-employed and employed women had higher odds of malaria infection (See Additional file 1: Table S4).

**Table 6:** Association between maternal characteristics and malaria infection during last pregnancy among women who experienced a pregnancy outcome in the past year in three districts of Jimma Zone, Ethiopia, 2016^a^

	Malaria infection during last pregnancy N (%)	No malaria infection during last pregnancy N (%)	*P* value for the overall difference among the categories	Factors associated with malaria infection during last pregnancy, univariate analysis OR (95% CI)	Factors associated with malaria infection during last pregnancy, multivariable analysis AOR (95% CI)^g^
Maternal age (years)					
15–18	11 (20.0)	274 (7.3)	**0.0088**	Reference	Reference
19–24	14 (25.4)	951 (25.5)		**0.36 (0.16–0.80)**	0.48 (0.19–1.18)
25–34	23 (41.8)	1887 (50.6)		**0.29 (0.14–0.62)**	**0.25 (0.11–0.58)**
35–49	7 (12.7)	617 (16.5)		**0.28 (0.11–0.73)**	**0.21 (0.07–0.65)**
Employment status					
Not Employed^b^	31 (56.4)	2927 (78.5)	**0.0007**	Reference	Reference
Self-employed^c^	21 (38.2)	705 (18.9)		**2.97 (1.68–5.22)**	**2.81 (1.55–5.11)**
Employed	3 (5.4)	97 (2.6)		2.99 (0.87–10.17)	3.50 (0.99–12.38)
Education level					
No education	39 (70.9)	2062 (55.3)	0.0790	Reference	Reference
Primary^d^	11 (20.0)	1162 (31.2)		**0.48 (0.24–0.95)**	**0.42 (0.20–0.88)**
Secondary^e^ or higher^f^	5 (9.1)	505 (13.5)		0.48 (0.19–1.25)	0.42 (0.15–1.21)
ITN utilization during pregnancy					
No	42 (76.3)	2745 (73.6)	0.6999	Reference	Reference
Yes	13 (23.6)	984 (26.4)		0.83 (0.43–1.58)	0.87 (0.44–1.72)
Indoor Residual Spraying					
No	39 (70.9)	2784 (74.6)	0.5260	Reference	Reference
Yes	16 (29.1)	945 (25.3)		1.17 (0.62–2.23)	1.45 (0.73–2.90)

*Abbreviations*: *CI* Confidence interval, *OR* Odds ratio, *AOR* Adjusted odds ratio

Notes: ^a^ Numbers are rounded and may not add up to exactly 100%; ^b^ the unemployed category contains women who were unemployed, housewives or students; ^c^ the self-employed category includes women who are farmers and/or traders; ^d^ considers women who completed all or some primary school years; ^e^considers women who completed all or some secondary school years; ^f^secondary and higher education were combined given model conversion issues; ^g^ AOR and corresponding 95% CI were obtained from a multivariate model, including variables with a significant univariate test at *p*-value cut-off point of 0.05

Bold text indicates significant results at the level of *p*-value <0.05

## Discussion

Using data from a cross-sectional survey conducted with women who had a pregnancy outcome in the year preceding the survey in three rural districts of Jimma Zone, this study identified the levels and determinants of ANC attendance, the ownership and use of ITNs for the prevention of malaria in pregnancy, and the prevalence of malaria in pregnancy in the three districts.

Across the three districts, an estimated 84% of women reported attending at least one ANC visit. Our findings are similar to what Abosse et al. observed in Hadiya Zone, southern Ethiopia (86%), but higher than what was reported in other studies conducted in eastern and northern parts of Ethiopia [18–22]. Our findings are also higher than the 51% reported in the 2016 EDHS for Oromiya region [3, 7]. Geographical differences in ANC attendance may depend on physical proximity of communities to ANC services, and women’s cultural beliefs and socio-economic status. Access to ANC services may also represent a challenge if the road conditions are poor or if the distance to the nearest health facility presents a barrier. In contrast to the high proportion of women who attended at least one ANC visit, only 47% reported attending four or more ANC visits. This is higher than the national-level estimate of 32% reported by the DHS group [3]. In a study conducted in Jimma Zone a decade ago, about 76% of the women attended one ANC visit, while 6.5% attended the recommended four visits [23]. A more recent survey conducted in 6 districts of Jimma Zone highlighted that 93% of women attended ANC services at least once during their last pregnancy and 47% completed four ANC visits [24]. While clear improvements over time in the coverage of ANC attendance are observed in Jimma Zone, close to 16% of our sampled women did not seek ANC at all. Of that group of 16, 50% of the respondents believed that ANC was unnecessary. Although not assessed in this study, the low proportion of women seeking four or more ANC visits may be explained by women’s poor satisfaction with ANC services. A qualitative assessment performed in Jimma Zone reported that women are unsatisfied with the services received due to a lack of confidence in the providers (healthcare workers, medical students), overcrowding of facilities and long wait time [25]. Attending fewer than four ANC visits could also be explained by the fact that women sought ANC services at an advance stage in their pregnancy and delivered before completing four ANC visits. The majority of the women in our sample attended ANC services in their second trimester (83%). This pattern is similar to research reporting that Ethiopian women tend to initiate ANC services in the second trimester [23–28]. A synthesis of qualitative studies in Malawi, Kenya and Ghana suggested that reproductive uncertainties, interaction with health staff and direct and indirect cost of ANC may influence the timing of ANC initiation [29]. Another qualitative assessment conducted in Ethiopia highlighted that although women are aware of when to receive ANC services, many will not seek ANC until they can feel the baby move [30]. Our findings indicate that governmental and programmatic efforts are still required to improve women’s knowledge of the importance and benefits of ANC services, and to surmount barriers that prevent women to access ANC services, such as distance, road conditions, and lack of transport, and cost of transport.

Acknowledging that women who attend the recommended number of ANC visits are more inclined to deliver in the presence of a skilled birth attendant, seek postnatal care and have better pregnancy outcomes [1], we assessed the determinants of receiving four or more ANC visits. Education level and wealth status were positively associated with attending four or more ANC visits. Women who were able to make decisions about their healthcare by themselves or with their male partners were also more likely to seek ANC services four or more times during their last pregnancy. The results obtained in our analysis are in line with past studies [6, 20–22], and suggest that additional work and initiatives are needed to promote a more equitable access to ANC services, and to overcome financial and knowledge barriers to not seeking ANC services among the poorest and uneducated women.

The proportion of pregnant women who owned at least one ITN in this study was lower than the 70% observed in a study conducted within the same study area in 2015 by Birhanu and colleagues [14]. Beside targeting a different study population, consisting of household heads, the difference in the proportions of households owning an ITN between the two studies may result from the fact that the other study followed a recent net distribution [14]. The utilization of ITNs, however, is quite consistent between the two studies; it is also similar to a study carried out in 2007 in 531 households of Oromiya region, 30% of which were recruited in Jimma Zone, wherein the utilization of ITNs among pregnant women was 59% despite the fact that roughly 90% of women owned a mosquito net [31]. This suggests that the barriers to optimal ITN utilization among pregnant women living in Jimma Zone have persisted over time. Previous studies focusing on the barriers to effective malaria prevention have indicated that perceptions regarding the risk of malaria and the effectiveness of ITNs were among the most important reasons for not using a net [7]. As suggested by our subgroup analyses, the ownership of ITNs during pregnancy was higher among women living within the catchment area of PHCUs that are at an altitude below 2000 m, where malaria can potentially be more endemic. Interestingly, the utilization of ITNs by women who owned ITNs was very similar to the proportion estimated considering the entire sample (60.1% vs. 59.5% in Gomma, 51% vs. 50.6% in Kersa and 51.7% vs. 52.2% in Seka Chekorsa). This suggests that, even though women from malaria-endemic areas are more likely to receive an ITN, the utilization of ITNs throughout the pregnancy remained low. There is a possibility that women from areas below 2000 m do not perceive malaria as a risk. Additional research could help to identify barriers to net use by women who owned an ITN.

Our study identified a significant association between a woman’s ANC attendance and the ownership and usage of a ITN during her last pregnancy. This is supported by similar findings reported in Nigeria and Mali, suggesting that ANC represents an effective mean for the delivery of ITNs to pregnant women [10, 32]. The relationship between ANC attendance and owning an ITN was stronger in our sub-group analysis (considering women living at an altitude below 2000 m). ANC service providers in these PHCUs may be aware of the increased risk of malaria in their community or may have been previously engaged by mass ITN distribution campaigns. The positive associations between ANC attendance and ITN ownership and utilization, which has not been previously assessed in Ethiopia, suggest that it is important to ensure that ITNs are made available to women during their ANC visits. Still, to ensure that women have access to ITNs, it is important to first address the barriers to and predictors of antenatal care attendance that were determined in this study and other assessments.

HEWs, health development army members and other healthcare providers interacting closely with pregnant women can play an important role in identifying and reaching the non-users of ANC. Being able to distinguish the differences between users of ANC from the non-users may allow HEWs and HDA members to specifically target women during their outreach activities who are less likely to seek ANC. Additional research and programmatic efforts are required to improve the physical accessibility to ANC services.

We found that the prevalence of malaria infection was low in our sample of women who were pregnant in the preceding year. While this self-reported estimate is subject to recall or reporting biases and may be affected by seasonal variations in malaria transmission, and should therefore be interpreted with caution, we found that the majority of malaria cases were observed in women from malaria-endemic areas, although malaria prevalence was not significantly different between the groups (1.4% versus 1.7%, respectively). Our results concur with the prevalence of 2.3% described by Newman et al. in low transmission settings, with results from Asmamaw et al., and with general trends in malaria prevalence indicated in the Malaria Indicator Surveys (MIS) for areas below 2000 m [8, 33]. The prevalence of malaria was relatively higher than what was reported in the Health Management Information System, but lower than what Nega et al. found in South Ethiopia [12, 34] . The low prevalence of malaria detected in this study also supports current national policies against the use of IPTp in Ethiopia [5, 7]. We identified few predictive factors, which may be explained by the low prevalence and/or the insufficient sample size and power to detect key risk factors for malaria infection. Even though the risk of malaria in pregnancy is lower in Ethiopia, relative to some other African countries, the risk of rapid progression to severe malaria and associated consequences among infected individuals, is relatively high, given that pregnant women in Ethiopia are less likely to acquire temporary immunity due to the low malaria parasite exposure in their lifetime. It is therefore key to continue to improve women’s access to malaria control tools (ITNs and IRS) and strengthen malaria case management services [8].

One of the main strengths of our study was the use of a large sample size to assess our associations of interest. While the cross-sectional nature of the data did not allow us to explore causal relationships, we established associations that have not been assessed in the past in this specific area of Ethiopia, and reiterated some of the gaps that will need to be overcome to improve maternal health outcomes. We considered a wide range of predicting factors in our analyses. Residual confounding remains possible, however, and measurement of some indices using self-report is subject to biases as previously mentioned. Variables pertaining to women’s reproductive history may be particularly subject to reporting bias. Their association with ANC attendance should be interpreted cautiously. While women who refused to participate might have been less likely to use ANC services and/or sleep under an ITN, and may have been at greater risk of malaria, given the high response rate (i.e. 98.5%), this would not likely have substantially changed the estimates and the direction or strength of the associations.

## Conclusions

Data from a community-based cross-sectional survey suggest that ANC attendance – particularly women’s attendance at four or more ANC visits – and the ownership and utilization of ITNs by pregnant women could be improved in three rural districts of Jimma Zone, Ethiopia. The main factors that were positively associated with ANC attendance were higher education and wealth status, the woman’s ability to make decisions regarding their own healthcare, and the intendedness of pregnancy. This work also indicates the importance of addressing physical accessibility barriers to ANC services and improving the knowledge of pregnant women and their families on the importance of attending ANC. Our finding of a strong relationship between ANC attendance and the ownership and utilization of ITNs emphasizes the importance of reducing barriers to pregnant women’s attendance to ANC, not only to receive an ITN but also to be informed on its appropriate utilization. Additional work is needed to promote a more equitable access to ANC services, and to overcome financial and knowledge barriers to not seeking ANC services among the poorest and uneducated women.

## Supplementary information

**Additional file 1: Table S1.** Subgroup analysis for rate of self-reported malaria infection rate, and ownership and use of ITNs among women who experienced a pregnancy outcome in the past year in three districts of Jimma Zone, Ethiopia, 2016^1^. **Table S2.** Subgroup analysis for association between antenatal care attendance and the ownership of bed nets among women who experienced a pregnancy outcome in the past year in three districts of Jimma Zone, Ethiopia, 2016. **Table S3.** Subgroup analysis for association between antenatal care attendance and the utilization of bed nets among women who experienced a pregnancy outcome in the past year in three districts of Jimma Zone, Ethiopia, 2016. **Table S4.** Subgroup analyses for association between maternal characteristics and malaria infection during last pregnancy among women who experienced a pregnancy outcome in the past year in three districts of Jimma Zone, Ethiopia, 2016.

## Data Availability

The datasets used and/or analyzed during the current study are available from the corresponding author on reasonable request.

## Abbreviations

ANC: Antenatal care
AOR: Adjusted odds ratio
CI: confidence interval
DHS: Demographic Health Survey
EDHS: Ethiopian Demographic Health Survey
HEW: Health Extension Worker
IRS: Indoor residual spraying
ITN: Insecticide-treated net
OR: Odds ratio
PHCU: Primary Health Care Unit
