# Supplementary material for: Utilization of key preventive measures for pregnancy complications and malaria among women in Jimma Zone, Ethiopia
Source: BMC Public Health. 2019 Nov 4;19:1443. doi: 10.1186/s12889-019-7727-8 (PMC6827171; doi:10.1186/s12889-019-7727-8)
Supplement: Supplementary file 1 — Additional file 1: Table S1. Subgroup analysis for rate of self-reported malaria infection rate, and ownership and use of ITNs among women who experienced a pregnancy outcome in the past year in three districts of Jimma Zone, Ethiopia, 20161. Table S2. Subgroup analysis for association between antenatal care attendance and the ownership of bed nets among women who experienced a pregnancy outcome in the past year in three districts of Jimma Zone, Ethiopia, 2016. Table S3. Subgroup analysis for association between antenatal care attendance and the utilization of bed nets among women who experienced a pregnancy outcome in the past year in three districts of Jimma Zone, Ethiopia, 2016. Table S4. Subgroup analyses for association between maternal characteristics and malaria infection during last pregnancy among women who experienced a pregnancy outcome in the past year in three districts of Jimma Zone, Ethiopia, 2016. [file 12889_2019_7727_MOESM1_ESM.docx]

Table S1. Subgroup analysis for rate of self-reported malaria infection rate, and ownership and use of ITNs among women who experienced a pregnancy outcome in the past year in three districts of Jimma Zone, Ethiopia, 2016^1^

|  | **Total**  **N (%)** | **Gomma**  **(N=1402)** | **Kersa**  **(N=1110)** | **Seka Chekorsa (N=1272)** |
| --- | --- | --- | --- | --- |
|  |  | N (%) | N (%) | N (%) |
| **Malaria infection during pregnancy** |  |  |  |  |
| Yes | 38 (1.7) | 18 (2.0) | 8 (1.0) | 12 (2.5) |
| No | 2157 (98.3) | 901 (98.0) | 780 (99.0) | 476 (97.5) |
| **Bed net ownership** |  |  |  |  |
| Yes | 65.5 | 694 (75.5) | 449 (57.0) | 294 (60.2) |
| No | 34.5 | 225 (24.5) | 339 (43.0) | 194 (39.8) |
| **Bed net use** |  |  |  |  |
| Never | 784 (35.7) | 239 (26.0) | 348 (44.2) | 197 (40.4) |
| Sometimes | 260 (11.8) | 122 (13.3) | 76 (9.6) | 62 (12.7) |
| Often | 351 (16.0) | 140 (15.2) | 135 (17.1) | 76 (15.6) |
| Always | 1798 (36.4) | 417 (45.4) | 229 (29.1) | 152 (31.1) |

^1^ Numbers are rounded and may not add up to exactly 100%

Table S2. Subgroup analysis for association between antenatal care attendance and the ownership of bed nets among women who experienced a pregnancy outcome in the past year in three districts of Jimma Zone, Ethiopia, 2016

|  | Owned a net  N (%) | Did not own a net N (%) | P value for the overall difference among the categories | Association with owning an ITN, univariate analysis  OR (95% CI) | Association with owning an ITN, multivariable analysis  AOR (95% CI) * |
| --- | --- | --- | --- | --- | --- |
| **ANC attendance** |  |  |  |  |  |
| No | 139 (9.7) | 174 (22.9) | **< 0.0001** | Reference | Reference |
| At least once | 1298 (90.3) | 584 (77.1) |  | **2.48 (1.90 – 3.24)** | **2.41 (1.82 – 3.19)** |

Note: * Adjusting for main confounders: maternal age, ethnicity, education level, occupation status, wealth, household size and indoor residual spraying

Abbreviations: ANC – Antenatal care, CI – confidence interval, ITN – Insecticide-treated net , OR – Odds ratio, AOR – Adjusted odds ratio

**Table S3. Subgroup analysis for association between antenatal care attendance and the utilization of bed nets among women who experienced a pregnancy outcome in the past year in three districts of Jimma Zone, Ethiopia, 2016**

|  | Always used a net  N (%) | Did not always use a net N (%) | P value for the overall difference among the categories | Association with always using a mosquito net, univariate analysis  OR (95% CI) | Association with always using a mosquito net, multivariable analysis  AOR (95% CI)* |
| --- | --- | --- | --- | --- | --- |
| **ANC attendance** |  |  |  |  |  |
| None | 70 (8.7) | 241 (17.4) | **<0.0001** | Reference | Reference |
| At least once | 728 (91.2) | 1154 (82.6) |  | **1.89 (1.40 – 2.54)** | **1.75 (1.29 – 2.38)** |

* Adjusting for main confounders: maternal age, ethnicity, education level, occupation status, wealth, household size, indoor residual spraying

Abbreviations: ANC – Antenatal care, CI – confidence interval, ITN – Insecticide-treated net, OR – Odds ratio, AOR – Adjusted odds ratio

Table S4. Subgroup analyses for association between maternal characteristics and malaria infection during last pregnancy among women who experienced a pregnancy outcome in the past year in three districts of Jimma Zone, Ethiopia, 2016

|  | Malaria infection during last pregnancy  N (%) | No malaria infection during last pregnancy  N (%) | P value for the overall difference among the categories | Factors associated with malaria infection during last pregnancy, univariate analysis  OR (95% CI) | Factors associated with malaria infection during last pregnancy, multivariable analysis  AOR (95% CI)^5^ |
| --- | --- | --- | --- | --- | --- |
| **Maternal age (years)** |  |  |  |  |  |
| 15 -18 | 4 (10.2) | 134 (6.2) | 0.7085 | Reference | Reference |
| 19 - 24 | 10 (25.6) | 527 (24.4) |  | 0.63 (0.19 – 2.06) | 0.87 (0.25 – 2.98) |
| 25 - 34 | 19 (48.7) | 1124 (52.1) |  | 0.56 (0.19 – 1.69) | 0.59 (0.19 – 1.85) |
| 35 - 49 | 6 (15.4) | 371 (17.2) |  | 0.54 (0.15 – 1.96) | 0.51 (0.13 – 1.92) |
| **Employment status** |  |  |  |  |  |
| Not Employed^1^ | 23 (58.9) | 1708 (79.2) | **0.0002** | Reference | Reference |
| Self-employed | 13 (33.3) | 391 (18.1) |  | **2.48 (1.24 – 4.94)** | **2.36 (1.17 – 4.77)** |
| Employed | 3 (7.7) | 57 (2.6) |  | **3.8 (1.12 – 13.27)** | **4.04 (1.14 – 14.32)** |
| **Education level** |  |  |  |  |  |
| No education | 26 (66.6) | 1172 (54.3) | 0.388 | Reference | Reference |
| Primary^2^ | 8 (20.5) | 668 (30.9) |  | 0.53 (0.24 – 1.18) | 0.48 (0.21 – 1.11) |
| Secondary^3^ or higher^4^ | 5 (12.8) | 316 (14.6) |  | 0.69 (0.26 – 1.83) | 0.54 (0.19 – 1.57) |
| **ITN utilization during pregnancy** |  |  |  |  |  |
| No | 26 (66.7) | 1371 (63.6) | 0.7250 | Reference | Reference |
| Yes | 13 (33.3) | 785 (36.4) |  | 0.87 (0.44 – 1.71) | 0.94 (0.46 – 1.89) |
| **Indoor Residual Spraying** |  |  |  |  |  |
| No | 23 (58.9) | 1230 (57.1) | 0.7893 | Reference | Reference |
| Yes | 16 (41.0) | 926 (42.9) |  | 0.93 (0.48 – 1.78) | 1.00 (0.51 – 1.97) |

Abbreviations: CI – confidence interval, ITN – Insecticide-treated net OR – Odds ratio, AOR – Adjusted odds ratio

Notes: ^1^ the unemployed category contains women who were unemployed, housewives or students; ^2^ considers women who completed all or some primary school years; ^3^considers women who completed all or some secondary school years; ^4^secondary and higher education were combined given model conversion issues; ^5^ AOR and corresponding 95% CI were obtained from a multivariate model, including variables with a significant univariate test at p-value cut-off point of 0.05
